# Tuberculosis in Small Ruminants in Portugal: A Retrospective Laboratory-Based Study (2012–2023)

**DOI:** 10.3390/ani16121755

**Published:** 2026-06-06

**Authors:** Handreza Junqueira Cobra, Leonor Orge, Paula Mendonça, Paulo Carvalho, Madalena Vieira-Pinto

**Affiliations:** 1 Department of Veterinary Sciences, Universidade de Trás-os-Montes e Alto Douro, 5000-801 Vila Real, Portugal; 2 Animal and Veterinary Research Center (CECAV), Universidade de Trás-os-Montes e Alto Douro, 5000-801 Vila Real, Portugal; 3 Instituto Nacional de Investigação Agrária e Veterinária (INIAV), 2780-157 Oeiras, Portugal

**Keywords:** caprine tuberculosis, health surveillance, histopathology, *Mycobacterium caprae*, One Health, Portugal, small ruminants

## Abstract

Animal tuberculosis caused by mycobacteria of the *Mycobacterium tuberculosis* complex (MTBC) represents a significant challenge to both public and animal health. Although eradication programs focus predominantly on cattle, small ruminants, especially goats, may play a relevant role as reservoirs. This study aimed to characterize tuberculosis cases in small ruminants in Portugal diagnosed at the national reference laboratory for Tuberculosis (Instituto Nacional de Investigação Agrária e Veterinária) over the period from April 2012 to July 2023. In this study, samples from 79 animals suspected of TB (64 goats and 15 sheep) were analyzed by integrating histopathology; bacteriology; and, when applicable, PCR results, thereby allowing the distribution by species to be described, MTBC agents to be identified, and diagnostic agreement to be assessed. Of these samples, 29 positive cases (36.7%) were identified, all of them in goats (45.3% of goats tested), whereas no cases were confirmed in sheep. *Mycobacterium caprae* was the most frequently identified species (89.7%), followed by *Mycobacterium bovis* (10.3%). Geographic distribution was concentrated in the Alentejo (48.3%) and Norte (44.8%) regions, where 93.1% of cases occurred. Topographical analysis of lesions revealed respiratory tract involvement (lung and/or thoracic lymph nodes) in 82.7% of positive cases, with gross patterns including caseous/caseocalcified lesions (*n* = 11), necrosis (*n* = 9), and nodular granulomatous lesions (*n* = 10). Agreement between histopathology and bacteriology was 81.5%. Parasitic coinfections were observed in 24.1% of positive cases, complicating histopathological interpretation. The results of this study indicate goats, as opposed to sheep, as non-negligible reservoirs of TB, suggesting species-level differences that warrant targeted investigation. The predominance of respiratory lesions and the detection of extensive cavitary forms indicate potential aerogenous transmission with implications for multi-host systems. Diagnostic discordance and parasitic interference reinforce the need for combined testing methodologies. It is therefore advisable to consider the integration of goats into tuberculosis surveillance and control programs, with greater relevance in Alentejo and Norte.

## Simple Summary

Animal tuberculosis affects several species and may also have implications for public health. Although control programs mainly focus on cattle, small ruminants, especially goats, may also contribute to the maintenance of infection. In this study, we reviewed laboratory records from Portugal collected between 2012 and 2023 to describe tuberculosis cases in goats and sheep. Among 79 animals investigated, 29 were confirmed as positive, and all were goats. Most cases were detected in the Alentejo and Norte regions. Lesions were mainly located in the lungs and associated lymph nodes, suggesting that respiratory transmission may be important in goats. We also found that combining laboratory methods improved case detection, while parasitic lesions sometimes complicated diagnosis. These findings indicate that goats should receive greater attention in tuberculosis surveillance and control programs in Portugal, particularly in regions where cases were more frequent. This information may help improve disease monitoring and support a broader One Health approach linking animal and public health.

## 1. Introduction

Animal tuberculosis, caused by mycobacteria of the *Mycobacterium tuberculosis* complex (MTBC), remains a significant health problem worldwide despite the implementation of control and eradication programs [1,2]. In addition to its direct impact on animal health and livestock production, it is a multi-host infection with public health implications, and its persistence has been associated with extensive production systems, interspecies circulation of the agent, and limitations of surveillance systems based predominantly on the detection of suspected cases or slaughterhouse findings [2,3,4]. In this context, postmortem inspection and laboratory confirmation play a central role in generating reliable and comparable epidemiological evidence, in line with WOAH recommendations for the diagnosis of mammalian tuberculosis [5,6].

In 2023, the number of human tuberculosis cases attributed to *Mycobacterium bovis* and *Mycobacterium caprae* in the European Union remained higher than during the COVID-19 pandemic years, although this pattern should be interpreted cautiously in the broader context of post-pandemic changes in tuberculosis case reporting [7].

Recent evidence from Spain further supports the zoonotic relevance of *M. bovis* and *M. caprae*, including human cases reported during 2018–2022 and genomic evidence of animal–human transmission in Catalonia [8,9]. Although official surveillance and eradication programs remain focused mainly on cattle, growing evidence indicates that small ruminants may also be involved in the epidemiology of MTBC infection in multi-host settings [1,9]. Their limited inclusion in formal control systems may therefore contribute to an incomplete understanding of infection dynamics at the animal–human interface [3].

The epidemiological relevance of small ruminants becomes particularly important in regions with evidence of interspecies transmission and human impact. In Catalonia (Spain), the circulation of *M. bovis* and *M. caprae* has been documented at the animal production–human interface, and genomic analysis indicated a probable zoonotic origin in up to 12% of the human cases assessed, with the involvement of small ruminants in part of these cases [10]. These data support the need to reassess the relative contribution of goats and sheep to the persistence of MTBC in systems where multiple hosts coexist.

In small ruminants, an operational paradox remains: most confirmed cases of infection by *M. bovis* and *M. caprae* are detected a posteriori through suggestive lesions identified at slaughter during postmortem inspection, whereas in vivo testing and targeted surveillance in these species remain conditional on the epidemiological context [1]. This pattern of passive detection limits the understanding of whether these species act as domestic reservoirs or merely as spillover hosts, a distinction that depends on the degree of infection, the pattern of interspecies contacts, and the intrinsic susceptibility of each species [2,4,9].

In Portugal, the Bovine Tuberculosis Eradication Program does not systematically include small ruminants, restricting intervention to scenarios involving an epidemiological link with infected cattle or suspicions raised during postmortem inspection [11]. In this context, slaughterhouse surveillance plays a primary role as a component of passive case detection, which may underestimate the magnitude and distribution of infection, given its dependence on the macroscopic visibility of lesions and on operational factors inherent to postmortem inspection [5,6].

The international literature describes outbreaks and detections of tuberculosis in small ruminants in different countries, often identified in slaughterhouse settings or on mixed farms, reinforcing the need for targeted surveillance and integrated diagnostic approaches in these species [12,13,14,15,16,17,18]. Selected international examples are summarized in Supplementary Table S1.

In Portugal, however, tuberculosis in small ruminants remains less well characterized than in other European countries. A relevant episode reported in 2008 occurred on a farm with 127 goats of the indigenous Serrana breed, in the northeast of the country, with clinical progression compatible with chronic respiratory disease and high mortality. Necropsy revealed caseous and caseocalcified lesions, predominantly in the lungs and mediastinal lymph nodes, with detection of acid-fast bacilli and culture confirmation of *M. bovis*. Among the survivors, 82.8% reacted positively to the tuberculin skin test, culminating in the sanitary culling of the herd [19].

After the publication of these cases in 2008, no further cases in small ruminants were reported in the published Portuguese literature, despite the increase in European reports and the growing recognition of the zoonotic relevance of tuberculosis in these species. Critical gaps therefore remain in the Portuguese context, namely: (i) the geographical distribution and frequency of detection in goats and sheep; (ii) the proportion of lesional forms, respiratory/digestive; (iii) the identification of MTBC agents; (iv) the agreement between conventional methods (histopathology and bacteriology); and (v) the interference of endemic coinfections in histopathological interpretation.

Thus, the present study contributes to filling some of the identified scientific gaps regarding this topic. Accordingly, the aims of this study were to characterize laboratory-confirmed tuberculosis cases in small ruminants in Portugal during the period 2012 to 2023; to describe patterns of distribution by species and region; to identify the predominant MTBC agents; to analyze lesion topography as an indirect indicator of transmission routes; and to evaluate the agreement between diagnostic techniques under passive surveillance conditions. These characterizations are essential for a One Health approach, in which structured surveillance on farms and at slaughterhouses is crucial for early detection, control, and mitigation of zoonotic risk [2,3,20,21], in line with WOAH guidance for the control of *M. tuberculosis* complex infection in livestock [22].

## 2. Materials and Methods

### 2.1. Study Design and Sample

The present study followed a retrospective and descriptive design, covering the results of samples from goats and sheep submitted through official veterinary channels for laboratory diagnosis of tuberculosis, based on the records of the National Institute for Agrarian and Veterinary Research (INIAV), which is the national reference laboratory for animal health. Submissions originated either from epidemiological control procedures or from suspicion identified during postmortem inspection at slaughterhouses. All submissions for suspected tuberculosis between April 2012 and July 2023 were considered. During this period, some temporal gaps were observed in the available records, consistent with periods without recorded laboratory submissions and/or absence of accessible documentation for analysis. However, this limitation does not affect the classification of the cases included.

During the study period, the results of analyses performed on samples from 79 animals (64 goats and 15 sheep) were evaluated at the INIAV laboratory. Sample processing generated 182 partial laboratory reports, issued throughout the diagnostic process (macroscopic examination, histopathology, bacteriology, and, when indicated, molecular identification by PCR).

### 2.2. Data Source

The data used in this study were extracted from routine laboratory records of INIAV, Portugal, referring to samples submitted for the investigation of tuberculosis in small ruminants. The variables collected included administrative identifiers (request number and individual sample identifier), reason for submission, species, region of origin, sample type, dates of collection and processing, macroscopic description, when available, and the results of the techniques applied (histopathology, bacteriology, and, when indicated, PCR). Information extraction was based on an integrated analysis of the structured fields and the free-text descriptions contained in the laboratory reports, through systematic manual review of all available records. Internal INIAV management variables, with no possibility of identifying operators and/or farms, were treated as anonymized data and used exclusively for internal organization and traceability of the process, thereby ensuring confidentiality.

### 2.3. Study Variables and Descriptive Analysis

For the descriptive analysis, the variables considered were species (goats/sheep), region of origin (Portugal NUTS II: Norte, Centro, Lisboa e Vale do Tejo, Alentejo, and Algarve), the framework of submissions within the bovine tuberculosis eradication program (sanitary culling due to epidemiological linkage to positive cattle herds or suspicion identified during postmortem inspection at the slaughterhouse), anatomical topography of lesions, and the MTBC agent in positive cases. The presence of parasitic coinfections, when identified by histopathology, and, when reported, the differential diagnosis were also recorded.

Lesion topography was determined based on macroscopic information and, when applicable, histopathological information, considering lung, lymph nodes (with anatomical discrimination whenever possible), liver, spleen, uterus, and kidney. The initial classification of suspected lesions was based exclusively on the macroscopic findings recorded during the anatomopathological evaluation at INIAV and was kept distinct from the subsequent diagnostic classification obtained by histopathology and/or bacteriology. Based on the anatomical pattern of lesions, a descriptive classification of indirect indicators of the likely route of infection (respiratory, digestive, or indeterminate) was also performed, without any intention of causal inference.

Data analysis was descriptive and based on the calculation of absolute and relative frequencies, with the results presented in tables and figures. Given the retrospective nature of the study, the passive submission of cases, and the absence of denominator data, no inferential analyses or confidence intervals were calculated.

### 2.4. Laboratory Procedures

Samples were processed according to the standardized procedures of INIAV. Histopathological; bacteriological; and, when indicated, molecular findings were integrated for the final assessment. Differential diagnoses suggested by the macroscopic and histopathological findings, including lesions compatible with caseous lymphadenitis, were recorded during routine laboratory evaluation, and Ziehl–Neelsen staining was used when appropriate to support the interpretation of granulomatous lesions.

#### 2.4.1. Macroscopic Examination

**Figure 1:**
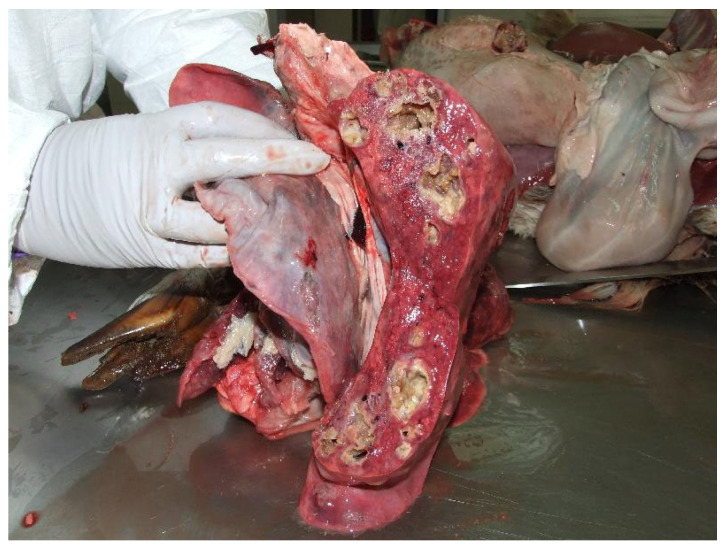
Macroscopic lesions compatible with tuberculosis observed in lung tissue of a small ruminant, characterized by multiple caseous nodules with areas of mineralization. Image provided by INIAV.

At INIAV, visual inspection of the submitted material was performed for macroscopic description of the lesions (Figure 1).

#### 2.4.2. Histopathology

After macroscopic evaluation, representative tissue fragments were selected for histopathology and bacteriology.

**Figure 2:**
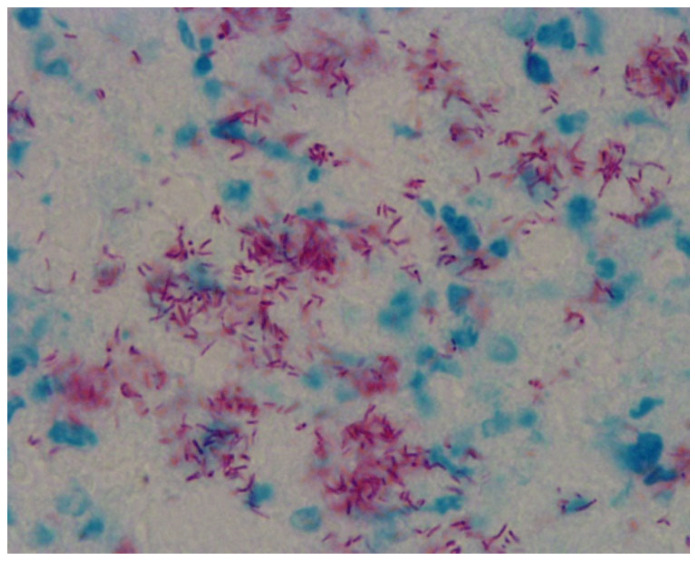
Ziehl–Neelsen staining demonstrating acid-fast bacilli (AFB) in lung tissue sample from a small ruminant, compatible with mycobacterial infection. Acid-fast bacilli stain red to fuchsia, whereas the background tissue and non-acid-fast elements appear blue. Image provided by INIAV.

Histopathological evaluation was performed on tissues fixed in 10% buffered formalin and processed using conventional histological techniques (paraffin embedding, microtomy, and hematoxylin and eosin staining (H&E)), with decalcification sometimes required in samples showing extensive calcification; when morphology was compatible with tuberculosis, Ziehl–Neelsen staining was performed to investigate acid-fast bacilli (AFB) (Figure 2). Samples were classified as: (i) compatible with tuberculosis, when typical granulomas with central caseous necrosis and infiltrates of epithelioid cells, lymphocytes, and Langhans-type giant cells and/or AFB were observed; (ii) suspicious, when suggestive granulomatous lesions were present, but without AFB and/or with morphological features that did not allow other etiologies to be excluded (including parasitic ones); and (iii) negative, when no compatible lesions were observed or when the changes were attributed to other causes. Before the use of formic acid as a decalcifying agent, Ziehl–Neelsen staining could not be performed, and classification was based exclusively on morphology.

#### 2.4.3. Bacteriology

Bacteriological investigation included microbiological culture in specific media for mycobacteria (Löwenstein–Jensen and/or liquid media), with incubation according to the standardized INIAV protocols, typically for 6 to 12 weeks. Isolate identification was performed by conventional and/or molecular methods, including, when applicable, PCR for differentiation of MTBC species, namely *M. bovis* and *M. caprae*.

#### 2.4.4. Molecular Identification (PCR)

PCR was used as a complementary method only when clinically and/or technically indicated, namely in situations of inconclusive histopathology results or when faster confirmation of the presence of MTBC mycobacteria was intended. Molecular identification included real-time PCR for identification of the *M. tuberculosis* complex (MTBC) and *M. bovis*, as well as PCR for identification of *M. caprae*. PCR was not applied systematically to all samples.

### 2.5. Diagnostic Workflow and Integration of Results

**Figure 3:**
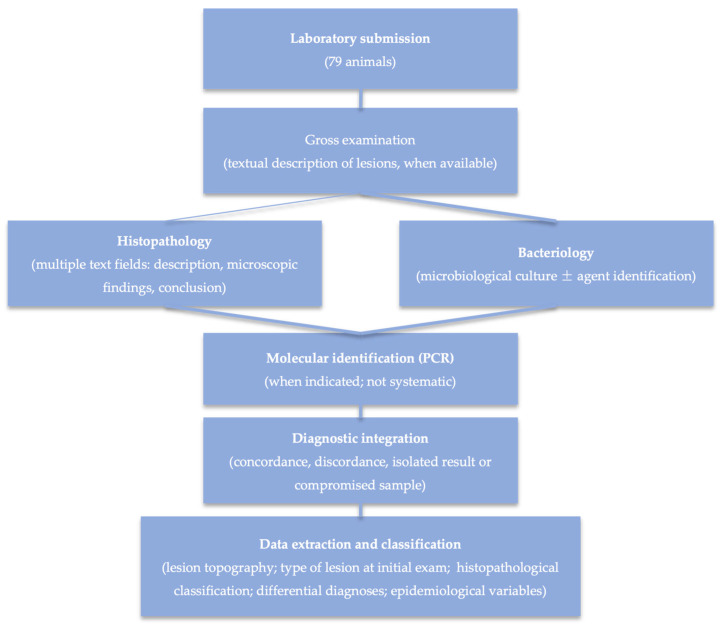
Diagnostic workflow applied to samples submitted for tuberculosis investigation.

The diagnostic workflow applied to samples submitted for tuberculosis investigation is summarized in Figure 3. The results were recorded in partial laboratory reports and integrated into a final classification for each sample, according to the following categories: (i) positive agreement (compatible/suspicious histopathology and bacteriological confirmation of MTBC mycobacteria); (ii) negative agreement (negative histopathology and absence of bacteriological isolation); (iii) disagreement between methods (negative histopathology with bacteriological confirmation, or compatible histopathology without bacteriological confirmation); (iv) single-method result (confirmation by only one method, with absence of a result from the other); and (v) compromised sample (impossibility of evaluation due to technical causes, including inadequate fixation, contamination, or advanced autolysis). These categories were used only to describe agreement between methods and result availability and did not correspond directly to the final classification of samples as MTBC-positive or MTBC-negative.

### 2.6. Ethical Considerations

This study was based exclusively on routine laboratory records from INIAV, the national reference laboratory for animal health, generated as part of regulatory animal health surveillance activities. No experimental manipulation of animals was performed. The data were anonymized, with no possibility of identifying farms or individual operators.

## 3. Results

### 3.1. Sample Population and Characterization of Submissions

During the period between April 2012 and July 2023, a total of 79 small ruminants were included in the study as suspected tuberculosis cases; most were goats (*n* = 64; 81.0%), whereas sheep were less represented (*n* = 15; 19.0%). Laboratory submissions originated mainly from sanitary culling within the bovine tuberculosis eradication program (*n* = 53), followed by suspicions identified during postmortem inspection at slaughter (*n* = 25) and necropsy (*n* = 1).

### 3.2. Bacteriological Identification and Distribution by Species

For the purposes of this study, a positive case was defined only as one with laboratory identification of MTBC agents by bacteriological culture and/or PCR, irrespective of the diagnostic workflow category. Based on this criterion, 29 positive cases were identified, corresponding to 36.7% (29/79) of the suspected animals; all positive cases occurred in goats. In sheep, no MTBC infection was confirmed (0/15; 0%), whereas in goats the positivity rate was 45.3% (29/64).

Regarding the reasons for submission among the positive cases (*n* = 29), 72.4% (*n* = 21) corresponded to sanitary culling within the bovine tuberculosis eradication program, 24.1% (*n* = 7) to suspicion identified during postmortem inspection at slaughter, and 3.4% (*n* = 1) to necropsy. Because the laboratory records did not include the total number of goats and sheep assessed within each submission route, these percentages refer only to the distribution of confirmed cases by route of submission and should not be interpreted as proportions of animals assessed within each route.

### 3.3. Geographical Distribution

The analyzed samples originated from different regions of mainland Portugal, with the highest proportion coming from the Norte (*n* = 31; 39.2%), followed by the Centro (*n* = 20; 25.3%) and Alentejo (*n* = 20; 25.3%), and a lower representation from Lisbon and Tagus Valley (*n* = 8; 10.1%). No samples were recorded from Algarve.

**Figure 4:**
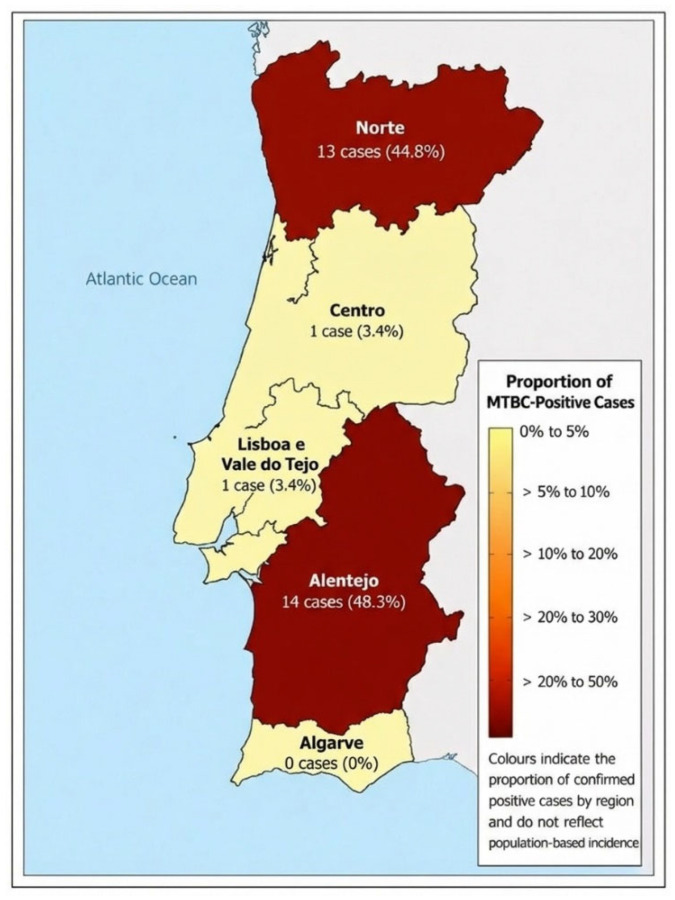
Regional distribution of laboratory-confirmed MTBC-positive cases in Portugal (2012–2023).

Among the positive cases (*n* = 29), the geographical distribution showed a higher concentration in Alentejo (*n* = 14; 48.28%) and in the Norte (*n* = 13; 44.83%), with one occurrence in the Centro (*n* = 1; 3.45%) and one in Lisbon and Tagus Valley (*n* = 1; 3.45%) (Figure 4). No occurrence was recorded in Algarve.

### 3.4. Identified MTBC Agents

Among the 29 positive cases, *M. caprae* predominated (*n* = 26; 89.7%), whereas *M. bovis* was identified in three cases (10.3%), two originating from Alentejo and one from Lisbon and Tagus Valley.

### 3.5. Temporal Distribution

The temporal distribution of submissions throughout the study period (2012–2023) was irregular, with records concentrated at specific time points and interspersed with prolonged intervals without submissions. Clusters of samples were observed in specific periods (e.g., April and December 2012; January and August 2013; October 2015; October and December 2016; August 2017; and several time points in 2023), a pattern consistent with the submission of multiple animals within the same episode of suspicion/investigation (detection at slaughter or within the bovine tuberculosis eradication program). This pattern is consistent with the passive nature of the system and with variation in detection/submission and in the level of detail available in the records over time; therefore, periods without records should not be interpreted as evidence of absence of disease.

A summary of the sample population and the main epidemiological findings is presented in Table 1.

### 3.6. Macroscopic Characterization of Lesions

Of the 29 MTBC-positive cases, macroscopic characterization was possible in 26; of these, 11 (37.9%) showed isolated pulmonary involvement; 13 (44.8%) showed pulmonary and corresponding lymph node involvement; and 2 (6.9%) had lesions restricted to lymph nodes, without evident macroscopic pulmonary involvement. In 3 cases (10.4%), useful macroscopic characterization could not be obtained because of lack of description or compromised evaluation.

Regarding the descriptive pattern of macroscopic lesions, the following changes were reported: necrotic lesions (*n* = 9), including extensive and coalescing areas, sometimes with irregular cavities (*n* = 2); caseous/caseocalcified lesions (*n* = 11); nodular granulomatous lesions (*n* = 10); and hemorrhagic changes (*n* = 8). In the lymph nodes, extensive caseous and caseocalcified lesions with central necrosis and mineralization were mainly recorded and, in some cases, nodular granulomatous lesions were also observed.

Detailed case-by-case characterization is available in the Supplementary Materials (Table S2).

### 3.7. Diagnostic Agreement

Agreement between histopathology and bacteriology was evaluated in the positive cases with both results available (*n* = 27), and agreement was observed in 22 cases (81.5%) and disagreement in five (18.5%), the latter corresponding to negative histopathology despite bacteriological confirmation.

### 3.8. Differential Diagnoses and Coinfections

Among the MTBC-negative goats (*n* = 35), the most frequent histopathological findings corresponded to the absence of lesions compatible with tuberculosis (*n* = 18; 51.4%), nonspecific pulmonary and/or lymph node lesions (*n* = 11; 31.4%), and lesions compatible with verminous pneumonia/parasitic granulomatous lesions (*n* = 6; 17.1%).

Among the MTBC-negative sheep (*n* = 15), the most frequent differential diagnoses were parasitic infections (*n* = 8; 53.3%), bacterial lymphadenitis (*n* = 6; 40.0%), and chronic bacterial pneumonia (*n* = 1; 6.7%).

Additionally, parasitic coinfections were recorded in 7/29 MTBC-positive cases (24.1%), mainly corresponding to verminous pneumonia and parasitic granulomatous lesions identified on pathological examination, which may complicate the interpretation of tuberculous-like lesions.

A summary of the main macroscopic, histopathological, and diagnostic findings is presented in Table 2.

## 4. Discussion

### 4.1. Main Findings and Epidemiological Interpretation

In this retrospective study, based on passive laboratory surveillance data collected between April 2012 and July 2023, infection by MTBC mycobacteria in small ruminants was confirmed exclusively in goats, with a predominance of *M. caprae*. In addition to the outbreak described in 2008 [19] on a goat farm in northeastern Portugal, associated with *M. bovis*, the present results add evidence on the involvement of small ruminants in the epidemiological dynamics of animal tuberculosis in Portugal.

As previously reported [5,6], postmortem inspection at slaughter played a relevant role in the detection of suspicions within passive surveillance, constituting the only route of submission in sheep and one of the routes of case identification in goats. In this study, sheep were submitted exclusively after suspicion during postmortem inspection, whereas goats also included animals subjected to sanitary culling within the bovine tuberculosis eradication program because of an epidemiological link with infected cattle herds. Thus, submissions after slaughter inspection reflected the incidental detection of suggestive lesions during routine postmortem inspection at the slaughterhouse, whereas submissions after sanitary culling reflected targeted investigation within the official eradication framework. Suggestive macroscopic lesions were identified in both species, although bacteriological confirmation of MTBC occurred exclusively in goats. This pattern is consistent with observations described in other European settings, in which goats assume greater epidemiological relevance in regions under Mediterranean influence (Spain, Italy, Greece), whereas sheep appear more frequently in distinct epidemiological contexts, described mainly in countries of Northern and Central Europe, such as the United Kingdom and Poland [12,13,14,16]. This geographical heterogeneity suggests the combined influence of multiple factors, including husbandry practices, intensity of interspecies contact, and differences in exposure dynamics.

The absence of confirmed cases in sheep should be interpreted with caution, given the passive nature of surveillance and the non-probabilistic nature of sampling. This pattern is consistent with the international literature, which describes greater detection of tuberculosis in goats in certain production systems and at interfaces with other species, without allowing inference of lower intrinsic susceptibility in sheep [23]. These hypotheses should be assessed in prospective studies with structured sampling and systematic antemortem testing, comparing both species in similar epidemiological contexts.

The predominance of *M. caprae* in confirmed cases places Portugal within the Iberian epidemiological context, where this species circulates in multi-host systems involving cattle, goats, and wildlife [5,14,18]. This pattern contrasts with that described in Italy, where *M. bovis* predominates in outbreaks in small ruminants [16]. Although fewer in number, cases of *M. bovis* were also identified in goats, indicating that more than one MTBC member was involved in the confirmed cases.

In the national epidemiological context, although *M. caprae* was identified predominantly in goats in the present study, its detection in cattle within slaughterhouse surveillance (7/381 of confirmed MTBC isolates; 1.8%) indicates circulation of the agent in more than one species at the national level [5]. Thus, the relative role of goats in the maintenance and transmission of the agent remains unclear, a question with direct implications for control strategies. To clarify transmission routes and interspecies relationships, molecular epidemiology approaches applied comparatively to isolates from goats, cattle, and wildlife will be particularly useful, as already demonstrated in Portugal for *M. bovis* in multi-host systems [24,25].

The officially bovine tuberculosis-free status recognized for Algarve since 2012 within the bovine tuberculosis eradication program may also have contributed to the absence of detected cases in that region, although the passive nature of surveillance does not allow underdetection in small ruminants to be excluded [11].

The concentration of positive cases in Alentejo and the Norte may reflect the action of multiple factors. These regions are characterized by extensive production systems, with greater contact among goats, cattle, and wildlife, and by a historically relevant presence of bovine tuberculosis within the bovine tuberculosis eradication program [11,26]. These factors may favor the maintenance and transmission of MTBC in multi-host systems [26]. The effect of passive surveillance intensity should also be considered, since these regions accounted for most of the submissions included in the study, which may have contributed to the higher detection of positive cases.

### 4.2. Lesion Pattern, Likely Transmission, and Diagnostic Performance

The predominance of thoracic lesions in the goats analyzed in this study, involving the lungs and thoracic lymph nodes, is consistent with respiratory infection, in agreement with natural and experimental studies describing caprine tuberculosis as a disease predominantly affecting the lower respiratory tract, with pulmonary and regional lymph node involvement [27,28]. This interpretation is descriptive, since the likely route of infection was inferred exclusively from lesion topography and not from direct demonstration of agent entry, although the predominance of respiratory involvement observed is consistent with aerosol transmission in contexts of close contact and cohabitation [12,16].

In nine MTBC-positive cases (31.0%), extensive pulmonary lesions with necrosis were observed, including, in two cases, irregular cavities, compatible with advanced-stage disease. In goats, this lesion pattern has been associated with higher bacillary load and greater potential for respiratory excretion, although confirmation of open forms of disease requires direct demonstration of the agent in respiratory samples (culture/PCR of secretions or bronchoalveolar lavage fluids) [3,27].

These findings also emphasize that not all granulomatous or caseous lesions observed in small ruminants are attributable to MTBC infection. In the present series, lesions compatible with verminous pneumonia, parasitic granulomatous inflammation, and caseous lymphadenitis were recorded among MTBC-negative animals and were also observed concomitantly with tuberculous-like lesions in some MTBC-positive cases. Such patterns may mimic tuberculous lesions at the gross or histopathological level and complicate pathological interpretation, particularly when necrosis, mineralization, and multinucleated giant cells are present. Pathogens and conditions contributing to this differential diagnosis include nematode-associated parasitic lesions and *Corynebacterium pseudotuberculosis* infection. Similar diagnostic challenges have been described in European goat studies, particularly in Spain, where concurrent paratuberculosis and caseous lymphadenitis have been reported as relevant sources of interference in tuberculosis diagnosis [29].

Among the 27 cases with both histopathology and bacteriology available, five discordant cases were observed (18.5%), indicating that histopathology alone might fail to detect a relevant proportion of infections confirmed by bacteriology [30,31]. In a surveillance context, in which diagnostic sensitivity is particularly relevant, this finding supports the use of combined approaches integrating histopathology and bacteriology, in agreement with international guidelines [22]. PCR is a useful complementary tool for the rapid resolution of inconclusive cases and direct confirmation of MTBC in tissue samples [12,22,32].

The differential diagnoses observed in MTBC-negative animals reinforce the limited specificity of passive surveillance based on suggestive lesions in small ruminants. In goats, verminous pneumonia/parasitic granulomatous lesions and nonspecific changes were frequent differential diagnoses, whereas in sheep, parasitic infections and bacterial lymphadenitis predominated [33,34].

The identification of parasitic coinfections in positive cases is a relevant finding, both for diagnosis and for the pathogenic interpretation of mycobacterial infection. Granulomatous lesions induced by pulmonary parasites, particularly *Muellerius capillaris*, may mimic tuberculosis both macroscopically and microscopically, especially in the absence of AFB [34]. This overlap constitutes a diagnostic challenge in regions endemic for parasitic infections because granulomatous pulmonary lesions in goats may correspond to tuberculosis, pulmonary verminosis, or the coexistence of both. Reliable distinction requires an integrated approach combining histopathology, parasitological investigation, and confirmation of MTBC by culture and/or direct PCR from tissue samples [22,30,33,35].

Taken together, these findings reinforce the relevance of health surveillance in extensive systems and at interspecies interfaces.

### 4.3. Implications for Surveillance and Control

Animals from which *M. bovis* was isolated (*n* = 3) originated exclusively from sanitary culling because of an epidemiological link. In the cases with histopathology available (*n* = 2), the lesion pattern was predominantly lymph node-related, with granulomas containing necrosis and/or calcification and identification of AFB, without macroscopic pulmonary lesions. This pattern suggests that the active component of the bovine tuberculosis eradication program, including screening, epidemiological investigation, and sanitary culling based on epidemiological linkage, may increase the detection of MTBC infections in goats, including cases that might not be identified by surveillance based only on macroscopic lesions observable at slaughter, whose sensitivity is limited [6,11].

In this context, surveillance targeted at goat farms with a history of bovine tuberculosis in the same production unit, in adjacent units, or with a recognized epidemiological link should be strengthened. The application of antemortem testing in these contexts may improve the monitoring of herds at increased risk, without diverting the existing focus of the bovine tuberculosis eradication program [11].

The concentration of confirmed cases in Alentejo and the Norte suggests that regions sharing similar epidemiological conditions, including extensive production systems and close livestock interfaces, should be prioritized for enhanced surveillance actions, without implying absence of infection in other areas where underdetection may exist [11].

Pilot screening projects in these areas may help establish baselines and define operational priorities. Strengthening surveillance in slaughterhouses with a higher volume of goat slaughter may also improve the consistency of submission of suspected cases and the quality of the information sent to the laboratory. Additionally, targeted cross-sectional studies on goat farms, particularly in extensive systems, may help to better estimate the magnitude of infection and identify higher-risk contexts.

Linking laboratory data with information from the bovine tuberculosis eradication program at the subregional level may support the identification of spatial overlaps and possible epidemiological links with foci in cattle. In areas with case clustering, analysis of the interspecies interface, complemented by molecular typing when available, may clarify epidemiological relationships and support risk-proportionate measures [23,24].

The effectiveness of passive surveillance depends on the ability of slaughterhouse inspectors and field veterinarians to recognize, describe, and submit suspected cases consistently. In small ruminants, this ability is particularly relevant because of the variability of lesion patterns and the lower operational familiarity with tuberculosis in this species [6].

Strengthening specific training in goats, with emphasis on the most frequent macroscopic patterns and atypical presentations, may improve the detection of suspected cases [6,29]. The availability of clear operational criteria for submission, accompanied by simple visual guides, may improve the quality and comparability of the information sent to the laboratory. Tuberculosis should be included in the differential diagnosis of chronic respiratory syndromes in goats, particularly in extensive systems [6]. Finally, regular feedback mechanisms for submitters, including summaries of results and of the main diagnostic disagreements, may support continuous learning and the progressive harmonization of practices.

Knowledge gaps remain that justify applied research to support surveillance operationally. Prevalence studies with probabilistic sampling in goat and sheep populations would allow estimation of the true burden of infection, which is still unknown. Case–control studies in goats may clarify risk factors, including husbandry practices, animal density, contact with cattle, and the occurrence of parasitic coinfections [24,34].

In parallel, the application of molecular typing, ideally by whole-genome sequencing, to isolates from goats, cattle, and wildlife in the same geographical areas may be crucial for reconstructing transmission networks and understanding the multi-host dynamics of infection. Comparative immunology research between goats and sheep could also contribute to clarifying possible differences in susceptibility between species. Finally, evaluation of the performance of antemortem tests in goats in the national context will be necessary to support more consistent screening protocols [24,34].

Tuberculosis in small ruminants occurs within multi-host systems and may have zoonotic implications, which reinforces the relevance of a One Health approach, particularly in areas where goats, cattle, and susceptible wildlife coexist and where integration among animal health surveillance, public health, and wildlife monitoring may support a more integrated epidemiological interpretation [3,7].

The aggregation and joint interpretation of laboratory information relating to goats with data from the bovine tuberculosis eradication program and, when available, with results from wildlife surveillance may support a more integrated interpretation of the spatial and temporal distribution of infection. For this purpose, functional mechanisms for information sharing among competent authorities and other relevant stakeholders may facilitate the timely detection of case clusters and the implementation of coordinated responses [4].

This framework should remain aligned with applicable European and international guidelines on zoonotic tuberculosis, ensuring strategic coherence and comparability among surveillance systems [2,3,7].

## 5. Study Limitations

The present study is based on passive surveillance data obtained from routine laboratory records, which introduces a selection bias inherent to case detection based on clinical suspicion, postmortem inspection findings, and epidemiological linkage to bovine tuberculosis foci. Consequently, the animals investigated tend to represent infections with more advanced lesional expression, not allowing inference regarding the occurrence of subclinical infections or estimation of population prevalence.

The absence of population denominators, namely goat and sheep populations by region, the number of animals slaughtered and inspected, and the submission rates of suspected cases, prevented the calculation of adjusted epidemiological indicators and limited comparisons between regions. Thus, the regional differences observed should be interpreted as surveillance information and not as measures of relative risk.

There are also diagnostic limitations. PCR was used only as a complementary and non-systematic method, which may have contributed to underdetection of cases in situations of negative or compromised culture. The unavailability or inadequacy of histological material in some cases affected the assessment of agreement between methods. In addition, classification of the likely route of infection was based exclusively on lesion topography, constituting an indirect indicator rather than direct demonstration of the route of entry of the agent.

Finally, the retrospective nature of the study entailed documentary limitations, including macroscopic descriptions with variable levels of detail and unavailability of material for reevaluation or additional testing in all cases. Together with the limited sample size and the absence of a control group, these limitations precluded inferential analyses and the assessment of associations, namely between parasitic coinfections and infection by MTBC agents. Even so, they do not compromise the value of the study as a systematic characterization of laboratory surveillance data from a national reference laboratory over a prolonged period, constituting a solid basis for the refinement of surveillance strategies and for defining priorities for future research.

## 6. Conclusions

This retrospective study, based on laboratory surveillance in Portugal, identified a consistent pattern of MTBC infection in goats, with a predominance of *M. caprae*. Although no cases were confirmed in sheep in the present series, the limited number of ovine samples does not allow definitive conclusions regarding their epidemiological role. The predominance of respiratory involvement is consistent with the potential for airborne transmission in multi-host settings.

The diagnostic agreement between histopathology and bacteriology, as well as the occurrence of discordant cases and the presence of parasitic coinfections, highlight the limitations of conventional diagnostic methods when applied in isolation and support the usefulness of combined approaches in small ruminants.

Although the limitations inherent to passive surveillance do not allow causal inferences or estimates of population prevalence, the results provide evidence to support three main operational recommendations: (i) systematic integration of small ruminants, particularly goats, into national tuberculosis surveillance and control programs, with emphasis on regions with recognized epidemiological risk conditions; (ii) implementation of combined diagnostic protocols; and (iii) investment in molecular epidemiology to reconstruct transmission networks and elucidate the role of goats as domestic reservoirs in multi-host settings.

In the current European context of strengthening the One Health approach to zoonotic tuberculosis, the limited integration of small ruminants, particularly goats in extensive Mediterranean systems, into official surveillance systems constitutes a gap that deserves greater attention. Taken together, these findings reinforce the relevance of small ruminants, particularly goats, in the epidemiology of animal tuberculosis in Portugal and support the need for more integrated surveillance, targeted investigation, and evaluation of complementary detection strategies in the national context.

## Figures and Tables

**Table 1:** Characterization of the sample population and main epidemiological findings.

Domain	Variable	Result
Study population	Total small ruminants investigated	79
	Goats	64 (81.0%)
	Sheep	15 (19.0%)
Submission category (overall)	Sanitary culling within the bovine tuberculosis eradication program due to epidemiological linkage	53 (67.1%)
	Suspicion identified during postmortem inspection at slaughter	25 (31.6%)
	Necropsy	1 (1.3%)
Laboratory-confirmed MTBC cases	Total positive cases	29 (36.7%)
	Positive goats	29/64 (45.3%)
	Positive sheep	0/15 (0.0%)
Submission category among positive cases	Sanitary culling within the bovine tuberculosis eradication program due to epidemiological linkage	21 (72.4%)
	Suspicion identified during postmortem inspection at slaughter	7 (24.1%)
	Necropsy	1 (3.4%)
Geographical distribution (overall)	Norte	31 (39.2%)
	Centro	20 (25.3%)
	Alentejo	20 (25.3%)
	Lisbon and Tagus Valley	8 (10.1%)
	Algarve	0 (0.0%)
Geographical distribution of positive cases	Alentejo	14 (48.3%)
	Norte	13 (44.8%)
	Centro	1 (3.4%)
	Lisbon and Tagus Valley	1 (3.4%)
	Algarve	0 (0.0%)
Identified MTBC species among positive cases	*M. caprae*	26 (89.7%)
	*M. bovis*	3 (10.3%)

Abbreviations: MTBC, *Mycobacterium tuberculosis* complex. Notes: Percentages for the overall population were calculated using 79 animals as the denominator. Percentages for positive cases were calculated using 29 laboratory-confirmed cases as the denominator.

**Table 2:** Macroscopic, histopathological, and diagnostic findings in laboratory-confirmed MTBC-positive cases and differential diagnoses in MTBC-negative animals.

Domain	Variable	Result
MTBC-positive cases	Total positive cases	29
Macroscopic characterization	Cases with useful macroscopic characterization	26 (89.6%)
	Cases without useful macroscopic characterization	3 (10.4%)
Lesion topography in positive cases (*n* = 26)	Isolated pulmonary involvement	11 (37.9%)
	Pulmonary and corresponding lymph node involvement	13 (44.8%)
	Lesions restricted to lymph nodes, without evident macroscopic pulmonary involvement	2 (6.9%)
Respiratory pattern	Respiratory tract involvement among positive cases	24/29 (82.7%)
Main macroscopic changes	Caseous/caseocalcified lesions	11
	Necrotic lesions	9
	Irregular cavitary lesions	2
	Nodular granulomatous lesions	10
	Hemorrhagic changes	8
Main histopathological findings in positive cases	Caseous necrosis	17
	Mineralization/calcification	14
	Granulomas	12
	Langhans-type giant cells	11
	Verminous pneumonia/parasitic granulomatous lesions	7
	Fibrosis	6
Diagnostic agreement	Positive cases with both histopathology and bacteriology available	27
	Agreement between methods	22 (81.5%)
	Disagreement between methods	5 (18.5%)
	Pattern of disagreement	Negative histopathology despite bacteriological confirmation
Differential diagnoses in MTBC-negative goats (*n* = 35)	Absence of lesions compatible with tuberculosis	18 (51.4%)
	Nonspecific pulmonary and/or lymph node lesions	11 (31.4%)
	Verminous pneumonia/parasitic granulomatous lesions	6 (17.1%)
Differential diagnoses in MTBC-negative sheep (*n* = 15)	Parasitic infections	8 (53.3%)
	Bacterial lymphadenitis	6 (40.0%)
	Chronic bacterial pneumonia	1 (6.7%)
Coinfections	Parasitic coinfections among MTBC-positive cases	7/29 (24.1%)

Abbreviations: MTBC, *Mycobacterium tuberculosis* complex. Notes: Percentages in the lesion topography subsection were calculated using the 26 MTBC-positive cases with useful macroscopic characterization as the denominator. Percentages in the diagnostic agreement subsection were calculated using the 27 MTBC-positive cases with both histopathology and bacteriology results available as the denominator. Macroscopic and histopathological findings were not mutually exclusive, and individual cases could present more than one lesion pattern or microscopic finding. Histopathological evaluation was not available or representative in all positive cases.

## Data Availability

The data presented in this study are available on request from the corresponding author. The data are not publicly available because they derive from official surveillance laboratory records held by INIAV, the national reference laboratory, and are subject to confidentiality restrictions and institutional governance. Anonymized aggregated data may be made available upon justified request, subject to institutional approval.

## Supplementary Materials

The following supporting information can be downloaded at: https://www.mdpi.com/article/10.3390/ani16121755/s1, Table S1: International reports of tuberculosis in small ruminants(country-by-country synopsis); Table S2: Case-by-case lesion characterization of MTBC-positive cases, including submission category, identified MTBC agent and gross lesions. References cited in the Supplementary Materials are included in the main reference list.
