# Supplementary material for: Tuberculosis in Small Ruminants in Portugal: A Retrospective Laboratory-Based Study (2012–2023)
Source: Animals (Basel). 2026 Jun 6;16(12):1755. doi: 10.3390/ani16121755 (PMC13296306; doi:10.3390/ani16121755)
Supplement: Supplementary file 1 [file animals-16-01755-s001.zip › animals-4345806-supplementary.pdf]

animals

Supplementary Materials

Table S1: International examples of tuberculosis in small ruminants (country-by-country synopsis)

Country/region	Epidemiological context	Species	Key agent / finding	Main take-home message	Reference
Basque Country (Spain)	Mixed sheep–goat farm; index suspicion detected at the slaughterhouse	Sheep + goats	New *M. caprae* spoligotype (sb2737); ELISA markedly more sensitive than cellular tests in that outbreak	Supports targeted surveillance, integrated diagnostics, and optimization of testing tools in small ruminants	Juste *et al.*, 2023
Ethiopia	Pastoral settings: shared watering points and grazing areas (often shared with cattle)	Goats (± cattle interface)	Isolation of *M. tuberculosis* and non-tuberculous mycobacteria in goats	Suggests potential human–animal transmission and highlights transmission opportunities at shared water/grazing points	Kassa *et al.*, 2012
Italy	Mixed herds under poor sanitary conditions	Cattle + goats	High frequency of respiratory lesions in goats; susceptibility to *M. bovis*; potential airborne spread	Removing only tb-positive cattle may be insufficient; integrated control across species is needed	Zanardi *et al.*, 2013
Italy (Sicily)	Two outbreaks detected via slaughterhouse lesion finding and on-farm testing (SICT); mixed farming/wildlife interface; confirmatory diagnostics incl. histopathology/IHC	Goats (Farm A and B), plus cattle/ovine in mixed system (Farm B context)	*M. bovis* outbreaks in goats; Farm A culture confirmed with spoligotype SB0841; Farm B final diagnosis via histopathology/IHC despite negative culture	Illustrates multi-host ecosystem challenges, the role of slaughterhouse detection, and the need to optimize diagnostic + regulatory strategies in endemic settings	Di Marco Lo Presti *et al.*, 2022
Southern Poland	Complex diagnostic scenario with discordant test results	Sheep	Igra positive and tb-like lesions postmortem despite negative tuberculin and negative culture	Highlights limitations of conventional tests in sheep and supports combined/targeted screening strategies	Didkowska *et al.*, 2022
Spain	National/regional eradication programs + abattoir surveillance + wildlife postmortem; molecular typing (spoligotyping) across multiple hosts	Goats, cattle, wildlife (wild boar, red deer, fox) + few sheep	*M. caprae* widely distributed; large dataset of 791 isolates, epidemiology driven by caprine infections	Shows multi-host circulation and supports goats as a major driver/reservoir in Spain; highlights value of routine molecular typing for surveillance	Rodríguez *et al.*, 2011
Wales (UK), 2008	Postmortem detection during slaughterhouse inspection; risk linked to animal movements	Goats (golden guernsey)	Extensive lesions: *M. bovis* (spoligotype sb0140) confirmed	Reinforces the value of slaughterhouse surveillance and highlight the biosecurity risks associated with animal movement and trade	Crawshaw *et al.*, 2008

Table S2: Case-by-case characterization of laboratory-confirmed positive cases, including submission category, identified MTBC agent and gross lesions.

Id	Region	Submission category	Identified MTBC agent	Lung gross lesions	Lymph node gross lesions	Other organs	Remarks
1	Centro	Not informed in laboratory record	*M. caprae*	No description	No description	—	No description
2	Alentejo	Sanitary culling	*M. caprae*	Extensive tissue destruction; irregular cavities	Caseocalcified lesions	—	—
3	Alentejo	Sanitary culling	*M. caprae*	Caseocalcified lesions	Caseocalcified lesions (1 LN)	—	—
4	Alentejo	Sanitary culling	*M. caprae*	Extensive caseocalcified lesions	Extensive caseocalcified lesions	—	—
5	Alentejo	Sanitary culling	*M. caprae*	Small caseocalcified nodules	—	—	—
6	Alentejo	Sanitary culling	*M. caprae*	Caseocalcified lesions	Extensive caseocalcified lesions	—	—
7	Alentejo	Postmortem inspection	*M. caprae*	Extensive areas of necrosis	No lesions	Liver and uterus without lesions	—
8	Alentejo	Postmortem inspection	*M. caprae*	Extensive areas of necrosis	No lesions	Liver without lesions	—
9	Alentejo	Postmortem inspection	*M. caprae*	Extensive areas of necrosis	No lesions	Liver and uterus without lesions	—
10	Alentejo	Postmortem inspection	*M. caprae*	Extensive necrosis with crater formation	—	Liver and uterus without lesions	—
11	Alentejo	Postmortem inspection	*M. caprae*	Not submitted	Extensive calcified necrotic lesions	Liver and uterus without lesions	Lung not submitted
12	Alentejo	Postmortem inspection	*M. caprae*	Extensive areas of necrosis	No lesions	Liver and uterus without lesions	—
13	Alentejo	Postmortem inspection	*M. caprae*	Extensive areas of necrosis	No lesions	Liver without lesions	—
14	Norte	Sanitary culling	*M. caprae*	Diffuse hemorrhages	Confluent caseous granulomatous lesions	—	—
15	Norte	Sanitary culling	*M. caprae*	Caseous granulomatous lesions with hemorrhage	No lesions	—	—
16	Norte	Sanitary culling	*M. caprae*	Diffuse hemorrhages	Confluent caseous granulomatous lesions	—	—
17	Norte	Sanitary culling	*M. caprae*	Diffuse hemorrhages	No lesions	—	—
18	Norte	Sanitary culling	*M. caprae*	Hemorrhagic foci	Nodular granulomatous lesions	—	—
19	Norte	Sanitary culling	*M. caprae*	Hemorrhagic foci with miliary nodular lesions	No lesions	—	—
20	Norte	Sanitary culling	*M. caprae*	Hemorrhagic foci with nodular granulomatous lesions	Nodular granulomatous lesions	—	—
21	Norte	Sanitary culling	*M. caprae*	Nodular granulomatous lesions	Nodular granulomatous lesions	—	—
22	Norte	Sanitary culling	*M. caprae*	Nodular granulomatous lesions	Nodular granulomatous lesions	—	—

23	Norte	Sanitary culling	*M. caprae*	Hemorrhagic foci	No lesions	—	—
24	Alentejo	Sanitary culling	*M. bovis*	No lesions	Necrotic lesions (1 extensive LN)	—	—
25	Alentejo	Sanitary culling	*M. bovis*	—	Assessment impaired	—	Inadequate fixation
26	Norte	Sanitary culling	*M. caprae*	Caseocalcified granulomas	Caseocalcified granulomas	Spleen and liver with lesions	—
27	Norte	Sanitary culling	*M. caprae*	Caseocalcified lesions	Caseocalcified lesions	Spleen with lesions; liver without lesions	—
28	Norte	Sanitary culling	*M. caprae*	Caseocalcified granulomas	Caseocalcified granulomas	Liver with lesions	—
29	Lisboa e Vale do Tejo	Sanitary culling	*M. bovis*	No description	No description	—	No description

Submission category was standardized from the laboratory record as follows: sanitary culling = sanitary culling within the bovine tuberculosis eradication program; post-mortem inspection = suspicion identified during post-mortem inspection at slaughter; necropsy = sample submitted for necropsy. Identified MTBC agent refers to the identified *Mycobacterium* species in each laboratory-confirmed positive case.
